# Who stays for the after party? Examining predictors of exercise engagement during and after an 8‐week gym‐based body composition challenge

**DOI:** 10.1111/aphw.70110

**Published:** 2026-01-09

**Authors:** Celina R. Furman, Sarah C. Volz

**Affiliations:** ^1^ Department of Psychology University of Michigan Ann Arbor Michigan USA; ^2^ Strategix Management LLC Washington District of Columbia USA

**Keywords:** behavior change, exercise adherence, gym‐based interventions, motivation, physical activity

## Abstract

Gym‐based programming may be a useful strategy to help people re‐establish consistency in their exercise routines and support long‐term exercise engagement. However, the effectiveness of gym‐based programs for behavior change is understudied. Using a prospective observational design, this study examines changes to participants' workout class attendance during and after an 8‐week body composition challenge at three group fitness studios, as well as individual factors that may moderate behavior change. Ninety‐one individuals (82.4% female) who were enrolled in the challenge completed an online survey assessing key predictors of exercise behavior identified by prior physical activity research, including prior exercise engagement, instrumental beliefs, enjoyment motives, and integrated regulation. Class attendance data were provided by the studios for the 8 weeks before, during, and after the challenge. This study found that class attendance generally increased for all participants during the challenge, and especially for those who previously attended fewer classes per week. These increases generally were not sustained after the challenge, returning to the same or less than pre‐challenge attendance levels. However, declines in class attendance were attenuated by higher enjoyment motives. Findings provide initial insight into the effectiveness of a gym‐based body composition challenge for behavior change, suggesting that while such programs might temporarily increase exercise engagement, additional strategies may be needed to sustain behavior change after program completion. Future research using experimental designs is needed to support these findings and better understand who may benefit most from gym‐based body composition challenges and gym‐based programming more broadly.

## INTRODUCTION

Regular physical activity offers extensive physical and mental health benefits for people of all ages (U.S. Department of Health and Human Services [USDHHS], 2018), yet most people struggle to sustain the amount of physical activity recommended to attain these benefits. Recent estimates show that nearly 80% of adults in the United States do not meet recommended guidelines at any given time (USDHHS, 2018), with even fewer adults doing so as they age (Elgaddal et al. 2022). The high prevalence of inactivity, and the health and financial costs associated with it, underscores the continued need for strategies that promote long‐term exercise engagement.

One strategy that people might use to support their exercise routines is participation in gym‐based body composition challenges, which target a common health goal and often have structure, incentives, and encouragement to bolster one's motivation and increase exercise engagement. For example, an unpublished pilot study on 43 participants in an 8‐week gym‐based body composition challenge found that many participants had goals related to increasing motivation and accountability to exercise, or to more generally establish a healthy routine (see Supporting Information). A limited number of studies have evaluated the effectiveness of similar programs for behavior change, finding increases in exercise motivation, intentions, and engagement during one's participation (Furman & Rothman, 2020; Martinez et al., 2022). However, high attrition (Gudzune et al., 2015; McEvedy et al., 2017) and post‐program exercise declines (Furman & Rothman, 2020) suggest that these benefits are fleeting, and that people may decrease or stop their exercise when they do not experience anticipated goal progress (Hugh‐Jones et al., 2021). Nevertheless, fitness facilities offer a promising platform for behavior change interventions as they provide an environment where individuals can have regular contact with the intervention, and a constant setting that promotes behavioral adherence after intervention cessation. Furthermore, their accessibility to the general public may remove barriers associated with clinical interventions or randomized controlled trials (e.g., availability, transportation, trial duration, and restrictive eligibility criteria; Fogel, 2018; Naidoo et al., 2020; Nipp et al., 2019). These features warrant further study to determine the effectiveness of gym‐based body composition challenges for long‐term behavior change, and for whom this type of programming might be most effective.

### Psychological predictors of behavior change

Psychological factors, such as the various motives underlying one's exercise and engagement in a program, may influence a program's effectiveness for behavior change. Converging perspectives from predominant theories of motivation, including self‐determination theory (Deci & Ryan, 2008, 2012), expectancy‐value theory (Eccles & Wigfield, 2002, 2020), and identity‐based motivation theories (Caldwell et al., 2018; Lewis & Oyserman, 2016), posit that people are more likely to sustain engagement in behaviors that are inherently rewarding, such as those that are enjoyable (i.e., intrinsic motivation) or align with one's identity (i.e., integrated regulation). In support, various clinical interventions and randomized controlled trials have found small effects of intrinsic motivation and integrated regulation on exercise behavior (Geller et al., 2018; Husband et al., 2019; Ntoumanis et al., 2021; Rhodes et al., 2025; Sheeran et al., 2020).

Believing that a behavior is instrumental to one's personal goals (i.e., instrumental beliefs) may also motivate engagement (Deci & Ryan, 2008, 2012; Eccles & Wigfield, 2002, 2020), but there is uncertainty regarding the sustainability of these beliefs over time. For instance, dissatisfaction with one's goal progress may weaken instrumental beliefs and subsequent behavioral engagement (Rothman, 2000; Rothman et al., 2004). Additionally, certain goal‐associated outcomes (e.g., body composition change) may serve as external contingencies (i.e., controlled sources of motivation) that undermine inherent sources of motivation (Deci & Ryan, 2008, 2012). These are important considerations in the current study context, as gym‐based body composition challenges inherently focus on outcomes and often use competition and financial incentives to motivate increases in exercise behavior. Studies have also found various negative consequences associated with exercising for weight loss, including reduced motivation over time, lower body and goal satisfaction, and less exercise engagement (Fuller‐Tyszkiewicz et al., 2018; Furman et al., 2025; Furman & Rothman, 2020; Greenleaf & Rodriguez, 2020; Homan & Tylka, 2014; Segar et al., 2006; Segar et al., 2011). Taken together, strong instrumental beliefs regarding a program's efficacy for one's goals may initially increase exercise engagement but may not be sufficient to sustain behavior change long‐term, especially when those goals are focused on outcomes related to weight or body composition.

### Research overview and program information

To better understand behavioral outcomes of gym‐based body composition challenges, the current study surveyed members of three franchised group fitness studios^1^ in Minnesota who were participating in an eight‐week body composition challenge from mid‐January to mid‐March in 2024. The challenge involved a competition between members for the greatest change in body composition (i.e., choice of percent body fat loss or lean muscle mass gain), which was measured in studio at the start and end of the challenge via a supervised InBody® 270 body composition scan. At each fitness studio, cash prizes were awarded to the top three individuals in each category, with the largest cash prize being awarded to the individual in first place. To be eligible for prizes, the challenge required members to take at least three classes per week for six of the eight weeks. Participants who attended three classes per week were also entered into weekly raffles for prizes such as gift cards, experience packages, and themed gift baskets from local businesses. Each class was a 1‐h group fitness workout that was instructed by a certified group fitness instructor and incorporated a combination of full‐body dumbbell strength training and moderate‐vigorous intensity cardiovascular exercise. Thus, attending three weekly classes would be sufficient to meet the 2018 Physical Activity Guidelines (USDHHS, 2018).

The survey was completed at the start of the challenge to assess motivational factors associated with exercise and demographic information. Class attendance data were obtained from the group fitness studios for the 8 weeks before, during, and after the challenge as an objective measure of physical activity. Two primary objectives were tested.

First, we evaluated the effectiveness of the challenge by examining behavior change during and after the challenge. Consistent with patterns of behavior change found in other physical activity interventions, we expected that class attendance would increase during the challenge (H1) but would decrease after the challenge (H2). We also examined if changes to class attendance differed by one's baseline attendance (i.e., average pre‐challenge weekly class attendance; RQ1). Second, we evaluated if motivational factors assessed at the start of the challenge moderated behavior change during and after the challenge. Drawing from the aforementioned theories of motivation, it was hypothesized that instrumental beliefs—the extent to which one believed that participating in the challenge would yield desired outcomes—would be associated with greater increases in class attendance during the challenge (H3), but that enjoyment motives and integrated regulation–two key predictors of exercise adherence–would attenuate declines in class attendance after the challenge (H4a and H4b, respectively).

## METHOD

This study was approved by the Institutional Review Board at the University of Michigan. For brevity, only variables pertinent to this paper's analyses are discussed in detail below. Complete materials, data, and analytic code are available on the Open Science Framework project page (https://osf.io/jk5dq/).

### Participants and protocol

On the first day of the challenge, members at the three participating fitness studios who were registered for the challenge (*n* = 210) were emailed information about the study with a link to the consent form and a Qualtrics survey including various measures of motivation, self‐efficacy, self‐reported physical activity, and demographic information (e.g., age, sex, race, and membership length). If interested, they were asked to complete the consent form and survey within 7 days. After completing the survey, the participants were entered into a random drawing to win a free month of membership to their studio.

Ninety‐one individuals (75 women; 82.4%) consented to participate and completed the survey. On average, the participants were 38.63 years old (*SD* = 10.18, range: 20–67) and reported being members at their fitness studio for 25.87 months (*SD* = 19.79, range: 0–83). Eighty‐one participants identified as Caucasian, non‐Hispanic; four identified as Asian or Asian American, non‐Hispanic, one identified as Mixed Race, non‐Hispanic; two identified as Caucasian, Hispanic; and three identified as Hispanic only.

### Measures

#### Exercise Motivation Inventory‐2 (EMI‐2)

The 4‐item enjoyment subscale from the EMI‐2 (Markland & Ingledew, 1997) was used to assess enjoyment motives for exercise. The participants indicated the extent to which they personally exercise for different reasons (“For the enjoyment of the experience of exercising”) on a 6‐point scale from 0 (not at all true for me) to 5 (very true for me) (*M* = 3.32, *SD* = 1.07, *α* = .88).

#### Behavioral Regulation in Exercise Questionnaire‐3 (BREQ‐3)

Integrated regulation refers to the extent to which one has integrated a behavior (i.e., exercise) into one's identity or sense of self (Deci & Ryan, 2008, 2012). Thus, the 4‐item integrated regulation subscale from the BREQ‐3 (Markland & Tobin, 2004; Wilson et al., 2006) was used to assess exercise identity‐based motivation. The participants reported the reasons they generally engage in exercise (“I consider exercise part of my identity”) on the same 6‐point scale as described for the EMI‐2. This rating scale was adapted from the original BREQ‐3 measure, which uses a 5‐point scale from 0 to 4, to reduce participant burden (*M* = 3.52, *SD* = 1.10, *α* = .82).

#### Instrumental beliefs

Instrumental beliefs were assessed through one item, developed by the researchers specifically for the present study's context: “Taking class at least 3 times per week for the next eight weeks will increase the likelihood that I will achieve my primary goal for the challenge.” The participants rated their agreement on a 6‐point scale from “*Strongly Disagree*” to “*Strongly Agree*” (*M* = 4.58, *SD* = 1.50).

#### Class attendance

For consenting participants (*n* = 88), we received class attendance data from each fitness studio with the number of classes that were attended in the 8 weeks before, during, and after the challenge (24 weeks total). Classes could be attended at any franchise location nationwide and were recorded by the studios' booking software. There was no limit on the number of classes that could be attended within a week.

### Data analytic plan

We first assessed the data for normality and outliers. To assess behavior change, a series of multilevel regression models were conducted predicting weekly class attendance for the 8 weeks before, during, and after the challenge. Multilevel models were used to appropriately account for the within‐person correlation of class attendance observations across different time points (e.g., pre‐, during, and post‐challenge). A random intercept for each person's class attendance was included to account for any baseline differences in class attendance that might exist across participants. Sex, age, and self‐reported membership length were assessed as potential covariates, to be included in each of the primary analyses if their associations with weekly class attendance were significant (*p* < .05).

Effects of the challenge were first examined by comparing weekly class attendance during the challenge with attendance pre‐ and post‐challenge (H1 and H2, respectively). Accordingly, all models used the “during” challenge period as the reference category, as our primary research questions focused on changes in weekly class attendance leading into the challenge (pre‐to‐during) and out of the challenge (during‐to‐post). To examine residual changes in weekly class attendance from pre‐ to post‐challenge, models were re‐run with the pre‐challenge period as the reference category, allowing direct comparisons of post‐challenge attendance relative to pre‐challenge attendance.

These analyses were then extended to test whether changes in weekly class attendance were moderated by participants' baseline attendance, including interactions of challenge period with one's baseline attendance (i.e., average pre‐challenge weekly class attendance) (RQ1). Additional extensions examined whether changes in weekly class attendance were moderated by the three motivational factors: instrumental beliefs (H3), enjoyment motives (H4a), and integrated regulation (H4b). Although we had originally planned to test the three motivational factors in a single model, concerns about suppression effects (see Table A in the Supporting Information for correlations between motivational factors) and potential limitations related to the sample size led us to test each motivational factor in a separate model. The full model testing all motivational factors simultaneously is included in Table B in Supporting Information. Each motivational factor was mean centered prior to being entered in the models.

## RESULTS

Weekly class attendance during the 24‐week observation period (*n* observations = 2059) was normally distributed (Figure A in Supporting Information). Outliers were identified and replaced with missing values for integrated regulation (*n* = 1) and enjoyment (*n* = 2). Two participants were missing responses for instrumental beliefs. Sex, age, and self‐reported membership length were not significantly associated with weekly class attendance (*p*'s > .21; Table C in Supporting Information) and were thus not included in primary analyses.

### Did weekly class attendance increase during and after the challenge?

On average, participants attended 2.73 (*SD* = 1.43, range: 0–6.38) classes per week in the eight weeks before the challenge, 3.61 (*SD* = 1.37, range: 0.63–7.00) classes per week in the 8 weeks during the challenge, and 2.54 (*SD* = 1.45, range: 0–6.00) classes per week in the eight weeks after the challenge. Consistent with these descriptive patterns, the multilevel model (Table D; Figure B in Supporting Information) showed that weekly attendance was significantly higher during the challenge than both the pre‐challenge period (*B* = −0.87, *SE* = 0.07, *p* < .001, semipartial *R* ^2^ = 0.04 [0.02, 0.05]) and the post‐challenge period (*B* = −1.06, *SE* = 0.07, *p* < .001, semipartial *R* ^2^ = 0.06 [0.04, 0.08]). There was also a small but significant residual decline in attendance from the pre‐ to post‐challenge period (*B* = −0.19, *SE* = 0.07, *p* = .01, semipartial *R* ^2^ = 0.002 [0.00, 0.01]; Table E in Supporting Information).

### Were changes in attendance moderated by baseline attendance?

**FIGURE 1:**
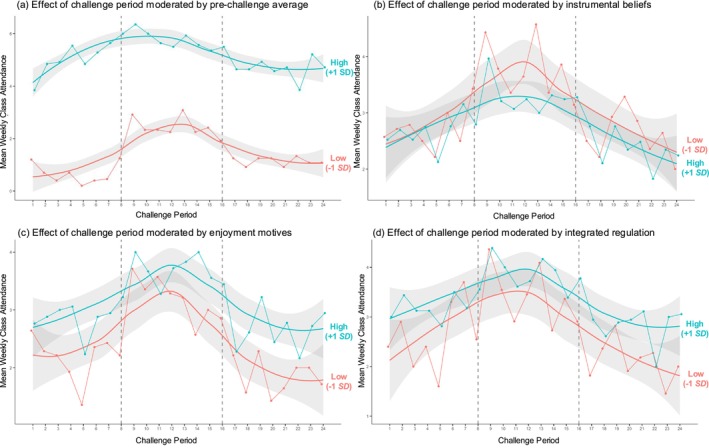
Effect of challenge period on weekly class attendance, moderated by (a) baseline class attendance (i.e., pre‐challenge average), (b) instrumental beliefs, (c) enjoyment motives, and (d) integrated regulation. The blue and red lines, respectively, represent higher (+1 SD) and lower (−1 SD) levels of each moderator. The dashed vertical lines indicate the final week of each challenge period.

Changes in class attendance based on challenge period remained significant when baseline attendance and its interaction with challenge period were entered into the model. As described in Table 1 (see Table F in Supporting Information for the model with pre‐challenge as the reference category), baseline attendance was associated with class attendance throughout the 24‐week observation period, such that those with higher (vs. lower) baseline attendance took more weekly classes before, during, and after the challenge. This effect was qualified by significant interactions between baseline attendance and challenge period (Figure 1a).

**TABLE 1:** Multilevel model predicting weekly class attendance based on baseline class attendance (i.e., baseline atn) and its interactions with each challenge period.

*Predictors*	*Estimates* [95% CI]	SE	*p*	*semipartial R* ^2^ [95% CI]
(Intercept)	3.61 [3.46, 3.75]	0.07	<.001	‐
Pre‐period	−0.88 [−1.02, −0.74]	0.07	<.001	0.06 [0.04, 0.08]
Post‐period	−1.06 [−1.20, −0.93]	0.07	<.001	0.09 [0.07, 0.12]
Baseline atn	0.70 [0.60, 0.80]	0.05	<.001	0.15 [0.12, 0.18]
Pre‐period*baseline atn	0.28 [0.19, 0.38]	0.05	<.001	0.01 [0.01, 0.03]
Post‐period*baseline atn	0.09 [−0.00, 0.19]	0.05	0.06	0.001 [0.00, 0.01]
*Random effects*				
*σ* ^2^	1.69			
*τ*_00 PID_	0.25			
ICC	0.13			
*N*_PID_	88			
Observations	2059			
Marginal *R* ^2^/conditional *R* ^2^	0.45/0.52			

*Note*: During challenge, class attendance is the reference group for challenge period.

Pairwise comparisons (see Table G in Supporting Information for estimated marginal means) indicated that weekly class attendance pre‐ and post‐challenge was lower than during the challenge for all participants, with the largest increases into the challenge and the largest decreases after the challenge occurring among participants with lower baseline attendance (during–pre‐estimate = 1.29, *SE* = 0.10, *p* < .001; during–post‐estimate = 1.19, SE = 0.10, *p* < .001) compared to those with higher baseline attendance (+1 SD; during–pre‐estimate = .47, SE = 0.10, *p* < .001; during–post‐estimate = 0.93, SE = 0.10, *p* < .001). Pairwise comparisons examining for residual changes in class attendance from pre‐ to post‐challenge indicated that pre‐ and post‐challenge class attendance did not differ significantly for those with lower baseline attendance (pre–post estimate = 0.09, *SE* = 0.10, *p* = .63), whereas post‐challenge attendance was significantly lower than pre‐challenge attendance for participants with higher baseline attendance (pre–post estimate = 0.46, *SE* = 0.10, *p* < .001).

### Were changes in attendance moderated by motivational factors?

The primary interest for Hypotheses 3, 4a, and 4b was to determine whether motivational factors moderated changes in weekly class attendance across challenge periods. Consistent with prior analyses, baseline attendance and its interaction with challenge period were included as covariates in all models.

#### Instrumental beliefs

As described in Table 2 and Figure 1b, instrumental beliefs did not have a main effect on class attendance throughout the 24‐week observation period and did not moderate changes in class attendance from pre‐challenge to during (not supporting H3), or during to post‐challenge. Residual changes in class attendance from pre‐ to post‐challenge also were not moderated by instrumental beliefs (Table H in Supporting Information).

**TABLE 2:** Multilevel model predicting weekly class attendance based on baseline class attendance (i.e., baseline atn), instrumental beliefs, and their interactions with each challenge period.

*Predictors*	*Estimates* [95% CI]	SE	*p*	*semipartial R* ^2^ [95% CI]
(Intercept)	3.59 [3.45, 3.73]	0.07	**<.001**	‐
Pre‐period	−0.86 [−1.00, −0.72]	0.07	**<.001**	0.06 [0.04, 0.08]
Post‐period	−1.05 [−1.19, −0.91]	0.07	**<.001**	0.09 [0.07, 0.11]
Baseline atn	0.70 [0.60, 0.80]	0.05	**<.001**	0.15 [0.12, 0.18]
Instrumental beliefs	−0.09 [−0.19, 0.01]	0.05	.08	0.003 [0.00, 0.01]
Pre‐period*baseline atn	0.28 [0.18, 0.38]	0.05	**<.001**	0.01 [0.01, 0.03]
Post‐period*baseline atn	0.09 [−0.00, 0.19]	0.05	.06	0.002 [0.00, 0.01]
Pre‐period*instrumental	0.08 [−0.02, 0.18]	0.05	.10	0.001 [0.00, 0.01]
Post‐period*instrumental	0.03 [−0.06, 0.13]	0.05	.50	0.00 [0.00, 0.003]
*Random effects*				
*σ* ^2^	1.70			
*τ*_00 PID_	0.25			
ICC	0.13			
*N*_PID_	86			
Observations	2011			
Marginal *R* ^2^/conditional *R* ^2^	0.45/0.52			

*Note*: During challenge, class attendance is the reference group for challenge period.

#### Enjoyment motives

As described in Table 3 and Figure 1c, enjoyment motives did not have a main effect on class attendance but did moderate changes in class attendance from during to post‐challenge. Pairwise comparisons (see Table I in Supporting Information for estimated marginal means) indicated that post‐challenge attendance was lower than during the challenge for all participants, with a steeper decline for participants with lower enjoyment motives (during–post‐estimate = 1.26, *SE* = 0.10, *p* < .001) than for those with higher enjoyment motives (during–post‐estimate = 0.93, *SE* = 0.10, *p* < .001; supporting H4a). Residual changes in class attendance from pre‐ to post‐challenge were not moderated by enjoyment (Table J in Supporting Information).

**TABLE 3:** Multilevel model predicting weekly class attendance based on baseline class attendance (i.e., baseline atn), enjoyment motives, and their interactions with each challenge period.

*Predictors*	*Estimates* [95% CI]	SE	*p*	*semipartial R* ^2^ [95% CI]
(Intercept)	3.63 [3.49, 3.78]	0.07	**<.001**	‐
Pre‐period	−0.91 [−1.05, −0.77]	0.07	**<.001**	0.07 [0.05, 0.09]
Post‐period	−1.09 [−1.22, −0.95]	0.07	**<.001**	0.10 [0.08, 0.12]
Baseline atn	0.71 [0.60, 0.81]	0.05	**<.001**	0.15 [0.12, 0.17]
Enjoyment	−0.06 [−0.20, 0.08]	0.07	.39	0.001 [0.00, 0.01]
Pre‐period*baseline atn	0.27 [0.17, 0.37]	0.05	**<.001**	0.01 [0.004, 0.02]
Post‐period*baseline atn	0.06 [−0.04, 0.16]	0.05	.25	0.001 [0.00, 0.01]
Pre‐period*enjoyment	0.07 [−0.06, 0.20]	0.07	.30	0.00 [0.00, 0.004]
Post‐period*enjoyment	0.16 [0.02, 0.29]	0.07	.**02**	0.002 [0.00, 0.01]
*Random effects* *σ* ^2^	1.63			
*τ*_00 PID_	0.25			
ICC	0.13			
*N*_PID_	86			
Observations	2011			
Marginal *R* ^2^/conditional *R* ^2^	0.45/0.52			

*Note*: During challenge, class attendance is the reference group for challenge period.

#### Integrated regulation

As described in Table 4 and Figure 1d, integrated regulation did not have a main effect on class attendance and did not moderate the change in class attendance from pre‐ to during, or during to post‐challenge (not supporting H4b). Residual changes in class attendance from pre‐ to post‐challenge also were not moderated by integrated regulation (Table K in Supporting Information).

**TABLE 4:** Multilevel model predicting weekly class attendance based on baseline class attendance (i.e., baseline atn), integrated regulation, and their interactions with each challenge period.

*Predictors*	*Estimates* [95% CI]	SE	*p*	*semipartial R* ^2^ [95% CI]
(Intercept)	3.60 [3.45, 3.74]	0.07	**<.001**	‐
Pre‐period	−0.87 [−1.01, −0.73]	0.07	**<.001**	0.06 [0.04, 0.08]
Post‐period	−1.07 [−1.20, −0.93]	0.07	**<.001**	0.09 [0.07, 0.12]
Baseline atn	0.68 [0.57, 0.78]	0.05	**<.001**	0.13 [0.10, 0.15]
Integrated regulation	0.04 [−0.10, 0.17]	0.07	.61	0.00 [0.00, 0.004]
Pre‐period*baseline atn	0.30 [0.19, 0.41]	0.05	**<.001**	0.01 [0.01, 0.03]
Post‐period*baseline atn	0.07 [−0.03, 0.17]	0.05	.17	0.001 [0.00, 0.01]
Pre‐period*integrated	−0.01 [−0.15, 0.12]	0.07	.85	0.00 [0.00, 0.003]
Post‐period*integrated	0.12 [−0.01, 0.25]	0.07	.08	0.001 [0.00, 0.01]
*Random effects*				
*σ* ^2^	1.70			
*τ*_00 PID_	0.25			
ICC	0.13			
*N*_PID_	87			
Observations	2035			
Marginal *R* ^2^/conditional *R* ^2^	0.43/ 0.51			

*Note*: During challenge, class attendance is the reference group for challenge period.

## DISCUSSION

The findings of this study provide preliminary insight into the behavioral outcomes of a gym‐based body composition challenge, both during and after one's participation. Study participants generally increased their weekly attendance during the challenge by about one class (i.e., 60 additional minutes of exercise), suggesting the challenge's initial effectiveness in boosting exercise frequency. However, the participants' class attendance generally declined after the challenge to the same or just below one's frequency of attendance before the challenge, aligning with patterns of behavior change observed in other physical activity interventions (Gasana et al., 2023; Howlett et al., 2019; McEwan et al., 2020; Murray et al., 2017; Yuan et al., 2024). Changes in class attendance during the study observation period were moderated by baseline attendance and enjoyment, a key motivational factor identified by prior theory and research (Deci & Ryan, 2012; Eccles & Wigfield, 2020; Geller et al., 2018; Husband et al., 2019; Ntoumanis et al., 2021; Rhodes et al., 2025; Sheeran et al., 2020).

### Variability in changes to class attendance by baseline activity level

Individuals who initially took fewer weekly classes (~1 class per week) showed the greatest changes in activity levels, increasing their class attendance to over two and a half weekly classes during the challenge. This finding highlights the challenge's potential in helping less active individuals increase their exercise frequency. However, post‐challenge declines suggest that less active individuals may require additional strategies to maintain initial increases to their class attendance and ensure the frequency of exercise recommended for substantial health benefits (i.e., ~3 weekly classes).

The challenge had a similar effect on individuals who previously were more regularly active within their fitness studios, such that those who averaged a greater number of weekly classes (~4 classes) pre‐challenge also increased their weekly attendance during the challenge (although to a lesser extent than their less active counterparts). Increases were not sustained after the challenge, but these individuals still attended more than three weekly classes, implying that they were maintaining the recommended amount of exercise for substantial health benefits through their group fitness studio.

Despite variability in the magnitude of change by baseline activity levels, unanimous increases in class attendance provide support for gym‐based body composition challenges as a feasible platform for interventions aiming to increase exercise engagement among individuals with varying activity levels. Aligning with other physical activity interventions (Gasana et al., 2023; Howlett et al., 2019; McEwan et al., 2020; Murray et al., 2017; Yuan et al., 2024), increases only occurred during the 8‐week challenge period. However, continued membership at one's gym may offer the opportunity for individuals to participate in various gym‐based programs throughout each year to recurrently boost their class attendance. Temporary increases to attendance should yield a generally higher level of physical activity over time and ultimately contribute to the broader goal of promoting lifelong health and well‐being. Research is needed to formally test if repeated participation in gym‐based programs can produce this effect, as well as identifying optimal times of the year for gyms to implement programs; for example, leveraging peak motivational periods such as after New Year's Day or during seasonal transitions to warmer weather (Ferguson et al., 2024; Garriga et al., 2021; Turrisi et al., 2021).

### Enjoyment attenuated post‐challenge declines in attendance

Although class attendance generally decreased for all participants after the challenge, the decline was less pronounced for those who reported higher enjoyment motives. This finding aligns with extensive research indicating that intrinsic motivation, such as exercising for enjoyment, is a key predictor of long‐term behavioral engagement (Eccles & Wigfield, 2002, 2020; Geller et al., 2018; Husband et al., 2019; Ntoumanis et al., 2021; Sheeran et al., 2020). Findings are also consistent with self‐determination theory, which posits that behaviors driven by intrinsic motivation are more likely to persist after external rewards—such as the competition and challenge incentives like weekly prize raffles and monetary rewards—are removed, because the behaviors themselves are inherently rewarding (Deci & Ryan, 2008, 2012). Taken together, gym‐based body composition challenges that foster enjoyment of exercise may help attenuate declines in engagement after program completion.

### Integrated regulation did not attenuate post‐challenge declines in class attendance

Like enjoyment, integrated regulation was expected attenuate declines in class attendance after the challenge; however, no evidence of moderation was observed. This contrasts with predominant motivation theories (Caldwell et al., 2018; Lewis & Oyserman, 2016), which suggest that individuals are more likely to sustain behaviors aligned with their identity or sense of self. Prior clinical interventions and randomized controlled trials have also found small effects of integrated regulation on exercise behavior (Geller et al., 2018; Husband et al., 2019; Ntoumanis et al., 2021; Rhodes et al., 2025; Sheeran et al., 2020). Notably, most prior research has targeted samples of inactive individuals who may have had lower baseline levels of integrated regulation. In contrast, our sample consisted largely of established gym members with moderate baseline integrated regulation, which may have reduced our study's ability to differentiate changes in class attendance during or after the challenge based on this motivational factor. Future research including less active participants who may have less integrated exercise identities may clarify the role of identity‐based motivation in sustaining exercise engagement after program completion.

Another potential explanation for the absence of moderation relates to the scope of measurement: our analyses focused solely on group fitness class attendance, which may provide only a partial picture of participants' overall activity. Individuals with strong exercise identities (or integrated regulation) may be more likely to engage in multiple forms of physical activity beyond structured classes. Thus, reduced class attendance may not necessarily indicate disengagement with overall exercise. Seasonal factors may also play a role in class frequency and activity type. For instance, physical activity volume has been found to increase with warmer weather (Ferguson et al., 2024; Garriga et al., 2021; Turrisi et al., 2021), which occurred during the post‐challenge period (March to May) in Minnesota, where the group fitness studios in this study were located. It is possible that participants attended fewer classes, which were indoor, to pursue more outdoor activities, such as trail running, hiking, cycling, or intramural sports. Assessing overall physical activity is needed to capture a more complete picture of how exercise routines shift in response to gym‐based body composition challenges and to examine potential moderating roles of motivational factors.

### Instrumental beliefs also did not moderate changes in class attendance

We hypothesized that instrumental beliefs would be associated with greater increases in class attendance during the challenge, as perceiving the challenge as important for achieving personal goals may motivate behavior (Deci & Ryan, 2008, 2012; Eccles & Wigfield, 2002, 2020). However, no moderation effect was observed. One possible explanation is that the challenge's focus on body composition may have attracted participants with weight‐related goals, which may have undermined both enjoyment of exercise and motivation to attend classes (Deci & Ryan, 2008, 2012). For instance, previous research suggests that focusing on weight loss or strongly believing that exercise will produce weight loss may have negative outcomes, including reduced motivation over time, lower body and goal satisfaction, and less exercise engagement (Fuller‐Tyszkiewicz et al., 2018; Furman et al., 2025; Furman & Rothman, 2020; Greenleaf & Rodriguez, 2020; Homan & Tylka, 2014; Segar et al., 2006, 2011).

Future studies should explore whether the reasons for program participation (e.g., body composition, general health, and behavioral consistency) influence the strength of association between instrumental beliefs and behavior. This is crucial to optimize the effectiveness of gym‐based programs more broadly, which often target body composition and use competition, financial incentives, or tangible prizes to enhance motivation. Shifting the focus from body composition to more proximal and achievable behavioral outcomes may be advantageous. For instance, a quasi‐experimental study on an eight‐week gym‐based fitness challenge found that people attended more workout classes during and after the challenge when they were instructed to focus on behavior (completing three classes per week) instead of weight loss, despite both challenges having the same workout requirements (Furman & Rothman, 2020). Experimental work is needed to test these ideas, especially as the gym‐based program's focus could contribute to self‐selection bias into the program and thus any related research study.

### Limitations and additional considerations

Generalizability of findings and inferences about causality are limited by the use of convenience sampling and the observational study design, respectively. In addition, the modest sample size may have limited the statistical power to detect smaller effects, particularly in analyses examining interactions with motivational factors or baseline attendance. Future research should use larger samples and experimental methods (both random sampling and experimental control) to address potential self‐selection bias and determine if observed increases to class attendance were because of the challenge itself, or some other variable, such as the challenge's close timing to the start of the New Year (occurring around Week 6 in the pre‐challenge observation period). For example, Figure 1 and Figure B in Supporting Information show that class attendance began to increase around week 6 in the pre‐challenge observation period and to decline around week 5 during the challenge (mid‐February). This temporal pattern aligns with research suggesting that people typically abandon New Year's resolutions sometime between 2 and 8 weeks (Dickson et al., 2021; Drive Research, n.d.; Norcross & Vangarelli, 1988).

To capture a more nuanced understanding of motivational dynamics and their impact on long‐term exercise engagement, future studies should also incorporate repeated assessments of motivational factors during and after the challenge. For example, time‐intensive assessments may better capture whether participating in a body composition challenge reduces exercise enjoyment, especially if one feels obligated to attend classes rather than chooses to do so willingly (Deci & Ryan, 2008, 2012). Additionally, satisfaction with goal progress or other exercise‐related outcomes may influence instrumental beliefs and motivation to continue one's exercise (Furman et al., 2025; Rothman, 2000; Rothman et al., 2004). This is especially relevant to the type of challenge evaluated in the current study, as many people underestimate the time and effort required to achieve body composition goals, which carries an inherent potential for unanticipated difficulties and setbacks (Kaftan & Freund, 2018; Polivy & Herman, 2002). Such unmet expectations could discourage continued exercise engagement that was initially adopted or increased in pursuit of body composition changes (Carmody et al., 1995; Scott et al., 2015; Sears & Stanton, 2001). Repeated assessments will also address the current study's limitation that motivational factors were only assessed at the start of the 8‐week challenge rather than concurrently with behavior throughout the challenge. Concurrent assessments will provide a more accurate depiction of the magnitude of the association between motivational factors and exercise adherence, and how it evolves over time.

Several aspects of our sample's demographic characteristics may also limit the applicability of these findings to broader populations. Study participants primarily identified as women, reflecting the largely female demographic typical of the group fitness environment in which the evaluated program occurred (The Exercise Movement and Dance Partnership, 2018). Importantly, research indicates that women are more likely than men to have weight dissatisfaction (Tsai et al., 2016) and to exercise for weight or aesthetic reasons (Craft et al., 2014; Hickey & Mason, 2017). As previously noted, these gender‐specific differences may have negatively affected how instrumental beliefs influenced class attendance, especially given that the evaluated program was a body composition challenge.

The participants were also primarily Caucasian, in early to mid‐adulthood, and likely had higher income levels, inferred from the studio membership cost (ranging from $140 to $170 each month). Additional research with larger representative samples is needed, especially given racial, economic, age, and gender disparities in physical activity (Centers for Disease Control and Prevention, 2017–2020; National Academies of Sciences, Engineering, & Medicine, 2017). Relatedly, this challenge was hosted by three group fitness studios with accountability structures (e.g., pre‐booking classes and late cancellation fees) and social support (e.g., fitness coaches and community building) that may not be available through other types of fitness facilities (e.g., box gyms and recreation centers). It will be important to evaluate the effectiveness of gym‐based programs in various types of facilities, and particularly in communities (e.g., low SES and rural) who may face access‐related barriers to exercise, such as financial and time restrictions, or limited access to specialized group fitness studios. Gym‐based programs in other settings and populations such as these may benefit from additional strategies to support long‐term exercise engagement. For example, the health action process approach highlights the importance of action and coping planning to identify and overcome potential barriers to exercise (Schwarzer, 2008). Prior work has also found that social interaction and support can facilitate the integration of exercise within one's social identity (see Stevens et al., 2017 for review) and may be particularly important facilitators of health behavior for adults living in low resource environments (Chang et al., 2008; MacFarlane et al., 2010; Thornton et al., 2006). These social factors are inherently built into group fitness environments but may need to be added to other gym‐based programs that do not involve group fitness.

Finally, we note that a large proportion of the current study's sample may have already been sustaining an exercise routine prior to the start of the challenge, as indicated by participants' average self‐reported membership length (~2 years) and pre‐challenge attendance (nearly 3 weekly classes). This detail helps us build on existing literature on exercise adherence, which is largely focused on identifying factors that encourage previously *inactive* individuals to increase their physical activity and sustain behavioral changes after intervention cessation (see Bernard et al., 2017; Howlett et al., 2019; Gasana et al., 2023; McEwan et al., 2020; Murray et al., 2017; Sheeran et al., 2016, 2020; Yuan et al., 2024 for recent meta‐analyses). A better understanding of exercise adherence in regularly active individuals is needed as maintaining an exercise routine may require one to overcome psychological and environmental barriers that might not affect new exercisers in the same way. For example, regularly active individuals might struggle with waning motivation, plateaued goal progress, or major life events (e.g., job and family transitions, moving, and injury/illness) that disrupt routines (Gropper et al., 2020). For these individuals, gym‐based body composition challenges or other gym‐based programs may be effective strategies to temporarily boost exercise engagement and help one re‐establish consistency in their exercise routine. The effectiveness of gym‐based body composition challenges for individuals who are initiating new exercise routines requires further examination, especially as these individuals often initiate exercise in pursuit of health and weight loss outcomes (Craft et al., 2014; Hickey & Mason, 2017) that may undermine the development of other key motivators of long‐term adherence (i.e., enjoyment and integrated regulation).

### CONCLUSIONS

Gym‐based programs represent a promising yet understudied avenue for physical activity interventions, offering the structure, encouragement, and consistent environment needed to facilitate long‐term exercise adherence. This study contributes to our understanding of the effectiveness of such programs, and identifies for whom they might be most beneficial. Although our evaluation focused on a specific 8‐week body composition challenge at three group fitness studios, findings have broader implications for leveraging gym‐based programs to reach and positively impact a wide audience. Given the global rise in the popularity of group fitness training (Thompson, 2018), refining such programs to promote inherently rewarding exercise experiences—such as focusing on enjoyment rather than body composition—remains a worthwhile pursuit. Follow‐up experimental research is essential to optimize these programs, ultimately ensuring they are effective tools for health behavior change and improved public health outcomes.

## CONFLICT OF INTEREST STATEMENT

The first author is a former employee of one of the group fitness studios included in this study but was not employed at the time this research was conducted because of relocation. No compensation was received for conducting this work, and the fitness studios were not involved in any step of the research process (e.g., study design, analysis of data, and writing or approval of manuscript) aside from providing access for participant recruitment. The authors declare that they have no other conflicts of interest.

## ETHICS STATEMENT

This paper represents an original contribution not previously published or being considered for publication elsewhere.

## Supporting information


**Table A SM.** Correlations between baseline variables and average weekly class attendance during each challenge period. M (SD) are represented on the diagonal.
**Table B SM.** Multilevel model predicting weekly class attendance based on pre‐challenge weekly average class attendance, enjoyment motives, integrated regulation, instrumental beliefs, and their interactions with each challenge period. During challenge class attendance is the reference group.
**Figure A SM.** Distribution of weekly class attendance across all 24 weeks (n = 2059 participant‐week observations). The x‐axis shows the number of classes attended per participant in a given week, and the y‐axis shows the frequency of participant‐weeks at each attendance level.
**Table C SM.** Results of multilevel model predicting weekly class attendance over the 24‐week observation period, with a random intercept for participant; used to test potential covariates (sex, age, and membership length).
**Figure B SM.** Weekly class attendance over time, with assessment periods indicated by color. Green represents the 8 weeks pre‐challenge, orange represents the 8‐week challenge period, and blue represents the 8 weeks post‐challenge.
**Table D SM.** Multilevel model predicting weekly class attendance based on challenge period. **During‐challenge class attendance is the reference group for challenge period.**
**Table E SM.** Multilevel model predicting weekly class attendance based on challenge period. **Pre‐challenge class attendance is the reference group for challenge period.**
**Table F SM.** Multilevel model predicting weekly class attendance based on baseline class attendance (i.e., baseline atn) and its interactions with each challenge period. **Pre‐challenge class attendance is the reference group for challenge period.**
**Table G SM.** Simple effects with estimated marginal means for each challenge period (i.e., before, during, and after) at low (‐1 SD) and high (+1 SD) baseline class attendance levels.
**Table H SM.** Multilevel model predicting weekly class attendance based on baseline class attendance, instrumental beliefs, and their interactions with each challenge period. **Pre‐challenge class attendance is the reference group for challenge period.**
**Table I SM.** Simple effects with estimated marginal means for each challenge period (i.e., before, during, and after) based on enjoyment motives.
**Table J SM.** Multilevel model predicting weekly class attendance based on baseline class attendance, enjoyment motives, and their interactions with each challenge period. **Pre‐challenge class attendance is the reference group for challenge period.**
**Table K SM.** Multilevel model predicting weekly class attendance based on pre‐challenge weekly average class attendance, integrated regulation, and their interactions with each challenge period. **Pre‐challenge class attendance is the reference group for challenge period.**

## Data Availability

Data and analytic code can be accessed on the OSF project page (https://osf.io/jk5dq/).

## ENDNOTES

^1^
The name of the group fitness studio franchise has been omitted from this paper upon ownership's request.
