# Supplementary material for: Who stays for the after party? Examining predictors of exercise engagement during and after an 8‐week gym‐based body composition challenge
Source: Appl Psychol Health Well Being. 2026 Jan 9;18(1):e70110. doi: 10.1111/aphw.70110 (PMC12789889; doi:10.1111/aphw.70110)
Supplement: Supplementary file 1 — Table A SM. Correlations between baseline variables and average weekly class attendance during each challenge period. M (SD) are represented on the diagonal. Table B SM. Multilevel model predicting weekly class attendance based on pre‐challenge weekly average class attendance, enjoyment motives, integrated regulation, instrumental beliefs, and their interactions with each challenge period. During challenge class attendance is the reference group. Figure A SM. Distribution of weekly class attendance across all 24 weeks (n = 2059 participant‐week observations). The x‐axis shows the number of classes attended per participant in a given week, and the y‐axis shows the frequency of participant‐weeks at each attendance level. Table C SM. Results of multilevel model predicting weekly class attendance over the 24‐week observation period, with a random intercept for participant; used to test potential covariates (sex, age, and membership length). Figure B SM. Weekly class attendance over time, with assessment periods indicated by color. Green represents the 8 weeks pre‐challenge, orange represents the 8‐week challenge period, and blue represents the 8 weeks post‐challenge. Table D SM. Multilevel model predicting weekly class attendance based on challenge period. During‐challenge class attendance is the reference group for challenge period. Table E SM. Multilevel model predicting weekly class attendance based on challenge period. Pre‐challenge class attendance is the reference group for challenge period. Table F SM. Multilevel model predicting weekly class attendance based on baseline class attendance (i.e., baseline atn) and its interactions with each challenge period. Pre‐challenge class attendance is the reference group for challenge period. Table G SM. Simple effects with estimated marginal means for each challenge period (i.e., before, during, and after) at low (‐1 SD) and high (+1 SD) baseline class attendance levels. Table H SM. Multilevel model predicting weekly class [file APHW-18-0-s001.docx]

**Supplementary Materials for**

**Who Stays for the After Party? Examining Exercise Adherence During and**

**After an 8-Week Gym-Based Body Composition Challenge**

**Table of Contents**

**Page 3:** Abbreviations used throughout SM

**Page 4:** Information about pilot study discussed in introduction and list of goals

**Page 5:** Table A SM. Correlations between motivational factors and class attendance during each challenge period

**Page 6:** Table B SM. Multilevel model predicting weekly class attendance based on pre-challenge weekly average class attendance, enjoyment motives, integrated regulation, instrumental beliefs, and their interactions with each challenge period

**Page 7:** Figure A SM. Distribution of weekly class attendance

**Page 7:** Table C SM. Multilevel model predicting weekly class attendance; testing sex, age, and membership length as potential covariates

**Page 8:** Figure B SM. Weekly class attendance over time

**Page 8:** Table D SM. Multilevel model predicting weekly class attendance based on challenge period (during-challenge as reference)

**Page 9:** Table E SM. Multilevel model predicting weekly class attendance based on challenge period (pre-challenge as reference)

**Page 9:** Table F SM. Multilevel model predicting changes in weekly class attendance based on baseline class attendance (pre-challenge as reference)

**Page 10:** Table G SM. Simple effects with estimated marginal means for each challenge period (i.e., before, during, and after) at low, mean, and high baseline class attendance levels

**Page 11:** Table H SM. Multilevel model predicting changes in weekly class attendance based on instrumental beliefs (pre-challenge as reference)

**Page 12:** Table I SM. Simple effects with estimated marginal means for each challenge period (i.e., before, during, and after) based on enjoyment motives

**Page 13:** Table J SM. Multilevel model predicting changes in weekly class attendance based on enjoyment motives (pre-challenge as reference)

**Page 14:** Table K SM. Multilevel model predicting changes in weekly class attendance based on integrated regulation(pre-challenge as reference)

**Abbreviations used throughout SM:**

Pre-period: 8 weeks before the challenge

Post-period: 8 weeks after the challenge

CI: 95% Confidence Interval

stdError: Standard Error

sp R² = semipartial R²

**Information about Pilot Study discussed in Introduction (2018, unpublished)**

Forty-three members of a group fitness studio who were participating in an 8-week weight loss challenge at their studio were surveyed about their goals for the challenge. These goals were coded for their inclusion of various outcomes (e.g., health, body composition, psychological benefits, motivation/accountability, fitness, lifestyle changes). Deidentified goals are included below.

| Boost energy, be healthier for my kids, fit into clothes better, feel better about self | increased strength and weight loss |
| --- | --- |
| Tone and gain more muscle mass | Lose 10 pounds in particular weight in my midsection. |
| To button my jeans. | Weight loss |
| get motivated to eat better to lose weight | Change body composition |
| Just fun to be in a competition where the benefits are better health | To lose weight and tone my muscles. |
| Weight loss | Prepare for triathlon season. |
| to work on fitness and nutrition at the same time | Lose body fat (2%) and a little body weight (5 lbs) |
| light a fire under my butt - I've been slacking and have gained some weight that I would like to get back off! | To learn about my body, develop a healthier and permanent lifestyle, and gain more energy to be successful in other areas of my personal life. |
| Accountability & motivation to maintain 4 workouts per week | To lose weight, lower body fat percentage and gain muscle tone. |
| Tone up and get stronger | Lose Weight - To get into my healthy weight soon and figure out a way to stay there. I have been going up and down in my weight ever since I was in my 20s |
| Lose weight, focus on healthy eating and sleep. | Strengthen, lose weight, tone |
| Weight loss and getting in a routine | Increase fitness, tone, health and decrease weight |
| I would like to increase my fitness, change my eating habits and lose some weight that I put on over the silly season! | To learn better eating habits and to focus my training on a few specific body areas. |
| Lose a little weight | Lose 10 lbs of fat |
| Losing weight, gaining more muscle | Accountability to exercise |
| Improved fitness through better food and beverage choices, plus stepping up my sessions for more impact. Expect that additional group accountability will be helpful as I've gained more weight over the winter than I want, which has bad long-term health effects. | I would like accountability. I was most drawn to the challenge because I will have a coach following up with me if I skip classes. I also have a friend doing it and enjoy feeling part of a team. Healthwise I would like to decrease my body fat % |
| Work out 3-4 times a week. This is also motivation to sort out how and what I want to eat in my life, because I’ve gone through a bunch of different things. I want to feel amazing in my body. | Lose 5 lbs and 4 inches. Improve my eating patterns. Better understand the drivers that affect my eating/snacking and how to make better decisions. |
| Lose weight, tone up, feel better, gain confidence | Weight loss Lean muscle and definition |
| Lose some baby weight. Get to a healthy weight. | To gain muscle and lose body fat. In addition to lose 7-10lbs. |
| Improve cardio health Commit to working out 3x/ week | Weight loss |
| to make a commitment to my health on a regular basis | Have a healthier outlook on my body image, increase self-confidence, tone up! |
|  | jumpstart my exercise program and try to stabilize my weight |

**Table A SM.** Correlations between baseline variables and average weekly class attendance during each challenge period. *M* (*SD*) are represented on the diagonal.

|  | 1 | 2 | 3 | 4 | 5 | 6 |
| --- | --- | --- | --- | --- | --- | --- |
| 1. Pre-challenge classes | 2.73 (1.43) |  |  |  |  |  |
| 2. Challenge classes | .73*** | 3.61 (1.37) |  |  |  |  |
| 3. Post-challenge classes | .78*** | .78*** | 2.54 (1.45) |  |  |  |
| 4. Instrumental beliefs | .05 | -.06 | -.12 | 4.58 (1.50) |  |  |
| 5. Enjoyment motives | .18 | .09 | .21 | .09 | 4.32 (1.07) |  |
| 6. Integrated regulation | .22* | .18 | .28** | -.08 | .66*** | 4.52 (1.10) |

*Note: *p < .05, **p < .01, ***p < .001*

**Table B SM.** Multilevel model predicting weekly class attendance based on pre-challenge weekly average class attendance, enjoyment motives, integrated regulation, instrumental beliefs, and their interactions with each challenge period. During challenge class attendance is the reference group.

| *Predictors* | *Estimates* | *Std. Error* | *95% CI* | *p* | *semipartial R*² |
| --- | --- | --- | --- | --- | --- |
| Intercept | 3.61 | .07 | [3.47, 3.76] | < .001 | - |
| Pre-period | -.89 | .07 | [-1.03, -.74] | < .001 | .06 |
| Post-period | -1.08 | .07 | [-1.22, -.94] | < .001 | .10 |
| Pre-class avg | .70 | .05 | [.59, .81] | < .001 | .45 |
| Enjoyment Motives | -.07 | .09 | [-.25, .11] | .46 | .001 |
| Integrated Regulation | .03 | .09 | [-.15, .21] | .76 | .00 |
| Instrumental Belief | -.07 | .05 | [-.17, .03] | .19 | .002 |
| Pre-period * Pre-class avg | .27 | .05 | [.17, .38] | < .001 | .01 |
| Post-period * Pre-class avg | .05 | .05 | [-.05, .15] | .32 | .00 |
| Pre-period * enjoyment | .06 | .09 | [-.12, .24] | .51 | .00 |
| Post-period * enjoyment | .09 | .09 | [-.09, .26] | .32 | .00 |
| Pre-period * integrated | .00 | .09 | [-.18, .18] | .97 | .00 |
| Post-period * integrated | .10 | .09 | [-.07, .27] | .25 | .00 |
| Pre-period * instrumental | .06 | .05 | [-.04, .16] | .22 | .001 |
| Post-period * instrumental | .01 | .05 | [-.08, .11] | .76 | .001 |
| *Random Effects* | | | | |  |
| σ^2^  τ_00_ _PID_  ICC  N _PID_ | 1.64  .25  .13  84 | | | |  |
| Observations | 1963 | | | |  |
| Marginal R^2^ / Conditional R^2^ | .45 / .52 | | | |  |

**Figure A SM.** Distribution of weekly class attendance across all 24 weeks (*n* = 2059 participant-week observations). The x-axis shows the number of classes attended per participant in a given week, and the y-axis shows the frequency of participant-weeks at each attendance level.


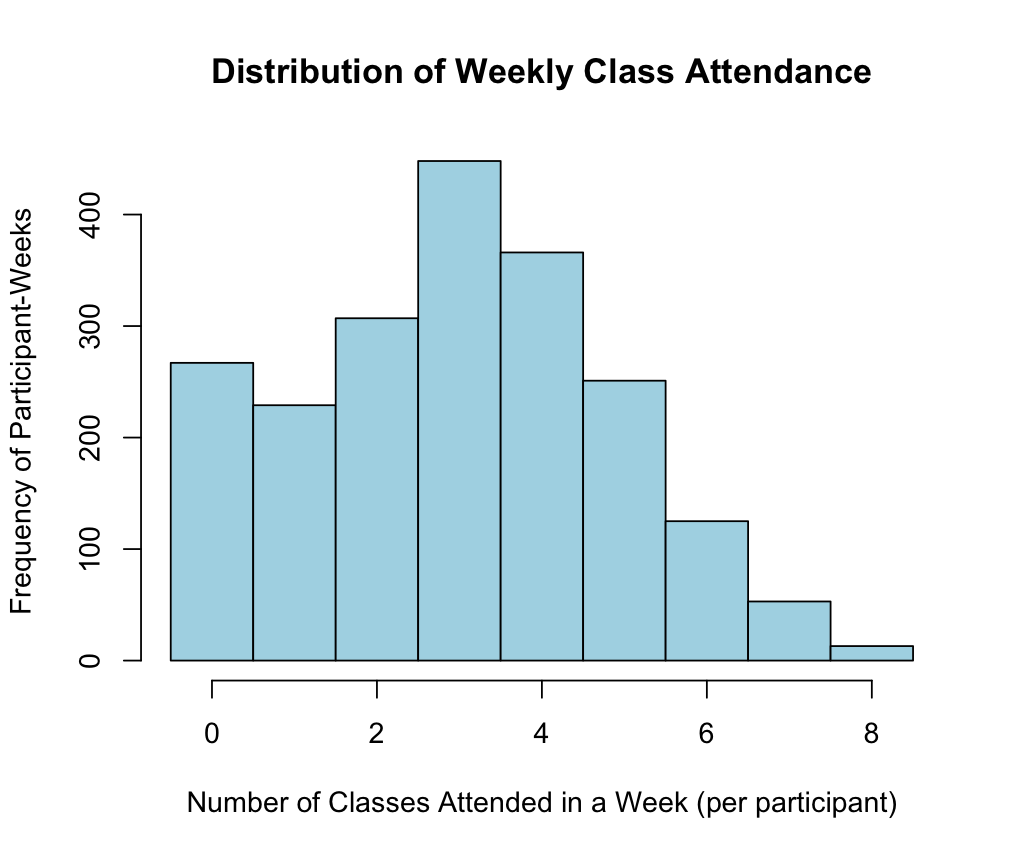


**Table C SM.** Results of multilevel model predicting weekly class attendance over the 24-week observation period, with a random intercept for participant; used to test potential covariates (sex, age, and membership length).

| *Predictors* | *Estimates [95% CI]* | | *std. Error* | *p* | *sp R² [95% CI]* |
| --- | --- | --- | --- | --- | --- |
| Intercept | 2.79 [1.60, 3.99] | | .61 | <.001 | - |
| Sex assigned at birth: | .21 [-.54, .96] | | .38 | .58 | .002 [.00, .01] |
| Age | -.00 [-.03, .03] | | .02 | .88 | .00 [.00, .003] |
| Membership length | .01 [-.01, .02] | | .01 | .21 | .01 [.003, .02] |
|  | |  |  |  |  |
| σ^2^  τ_00_ _PID_  ICC  N _PID_ |  | | | | |
| Observations  Marginal R^2^ / Conditional R^2^ |  | | | | |

**Figure B SM.** Weekly class attendance over time, with assessment periods indicated by color. Green represents the 8 weeks pre-challenge, orange represents the 8-week challenge period, and blue represents the 8 weeks post-challenge.

**
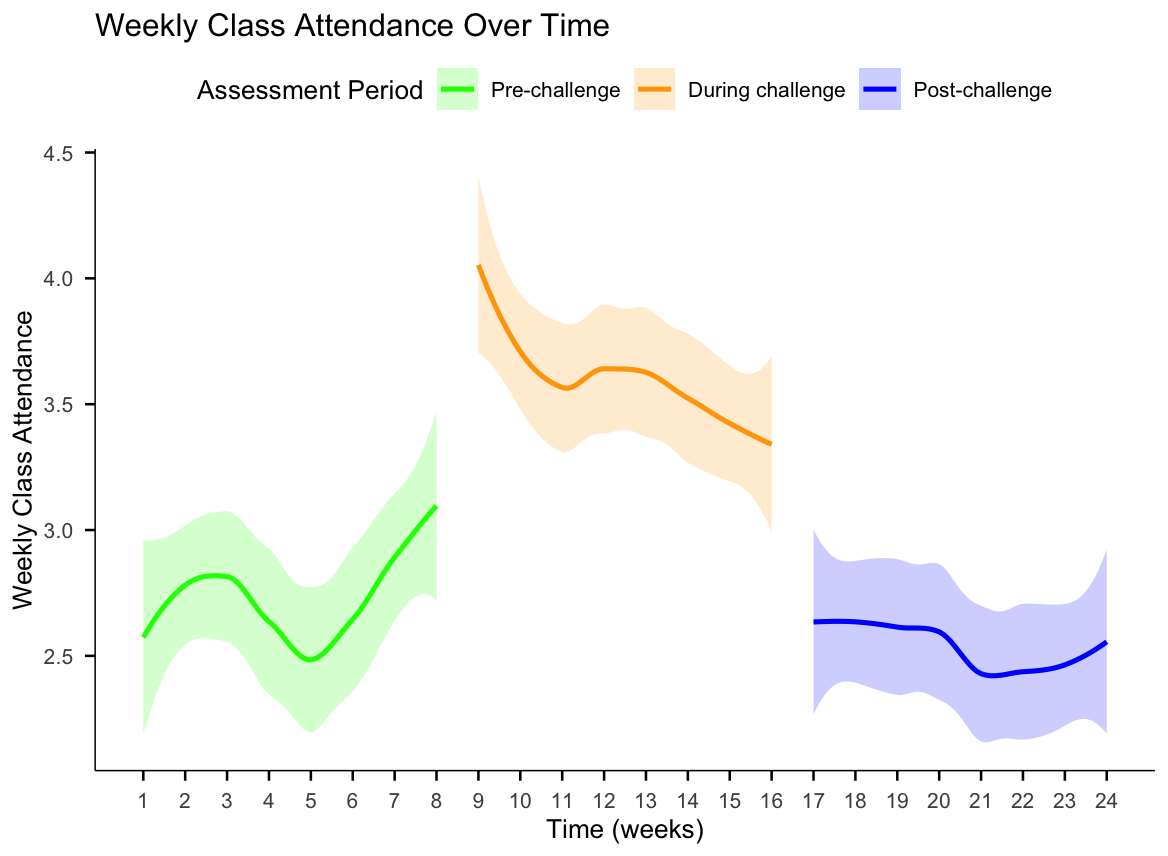
**

**Table D SM.** Multilevel model predicting weekly class attendance based on challenge period. **During-challenge class attendance is the reference group for challenge period.**

| *Predictors* | *Estimates [95% CI]* | *Std. Error* | | *p* | | *sp R*² *[95% CI]* |
| --- | --- | --- | --- | --- | --- | --- |
| Intercept | 3.61 [3.32, 3.89] | .14 | | < .001 | | - |
| Pre-period | -.87 [-1.01, -.73] | .07 | | < .001 | | .04 [.02, .05] |
| Post-period | -1.06 [-1.20, -.93] | .07 | | < .001 | | .06 [.04, .08] |
| σ^2^ | 1.71 |  |  | |  |  |
| τ_00_ _PID_ | 1.63 |  |  | |  |  |
| ICC | .49 |  |  | |  |  |
| N _PID_ | 88 |  |  | |  |  |

| Observations | 2059 |
| --- | --- |
| Marginal R^2^ / Conditional R^2^ | .06 / .52 |

**Table E SM.** Multilevel model predicting weekly class attendance based on challenge period. **Pre-challenge class attendance is the reference group for challenge period.**

| *Predictors* | *Estimates [95% CI]* | *Std. Error* | | *p* | | *sp R*² *[95% CI]* |
| --- | --- | --- | --- | --- | --- | --- |
| Intercept | 2.74 [2.45, 3.02] | .15 | | < .001 | | - |
| During-period | .87 [.73, 1.01] | .07 | | < .001 | | .04 [.02, .05] |
| Post-period | -.19 [-.33, -.05] | .07 | | .01 | | .002 [.00, .01] |
| σ^2^ | 1.71 |  |  | |  |  |
| τ_00_ _PID_ | 1.63 |  |  | |  |  |
| ICC | .49 |  |  | |  |  |
| N _PID_ | 88 |  |  | |  |  |

| Observations | 2059 |
| --- | --- |
| Marginal R^2^ / Conditional R^2^ | .06 / .52 |

**Table F SM.** Multilevel model predicting weekly class attendance based on baseline class attendance (i.e., baseline atn) and its interactions with each challenge period. **Pre-challenge class attendance is the reference group for challenge period.**

| *Predictors* | *Estimates* [95% CI] | SE | *p* | *semipartial R^2^* [95% CI] |
| --- | --- | --- | --- | --- |
| (Intercept) | 2.73 [2.58, 2.87] | .07 | <.001 | - |
| During-period | .88 [.74, 1.02] | .07 | <.001 | .06 [.04, .08] |
| Post-period | -.18 [-.32, -.04] | .07 | .01 | .003 [.00, .01] |
| Baseline atn | .98 [.88, 1.08] | .05 | <.001 | .24 [.21, .27] |
| During-period*Baseline atn | -.28 [-.38, -.19] | .05 | <.001 | .01 [.01, .03] |
| Post-period*Baseline atn | -.19 [-.29, -.10] | .05 | <.001 | .01 [.00, .02] |
| *Random Effects* |  |  |  |  |
| σ^2^ | 1.69 |  |  |  |
| τ_00_ _PID_ | .25 |  |  |  |
| ICC | .13 |  |  |  |
| N _PID_ | 88 |  |  |  |
| Observations | 2059 |  |  |  |
| Marginal R^2^ / Conditional R^2^ | .45 / 0.52 |  |  |  |

**Table G SM.** Simple effects with estimated marginal means for each challenge period (i.e., before, during, and after) at low (-1 SD) and high (+1 SD) baseline class attendance levels.

| Estimated Marginal Means | Estimated marginal mean | Std  Error | 95% CI |
| --- | --- | --- | --- |
| *Low baseline attendance (-1 SD)*  Pre-challenge classes  Challenge classes  Post-challenge classes | 1.32  2.61  1.42 | .11  .10  .10 | [1.12, 1.53]  [2.41, 2.81]  [1.22, 1.62] |
| *High baseline attendance (+1 SD)*  Pre-challenge classes  Challenge classes  Post-challenge classes | 4.13  4.60  3.67 | .10  .10  .10 | [3.92, 4.34]  [4.40, 4.81]  [3.47, 3.87] |
| Contrasts Based on Challenge Period | Estimate | Std  Error | *p** |
| *Low baseline attendance (-1 SD)*  During Challenge - Pre-challenge  During Challenge - Post-challenge  Pre-challenge - Post-challenge | 1.29  1.19  .09 | .10  .10  .10 | < .001  < .001  .63 |
| *High baseline attendance (+1 SD)*  During Challenge - Pre-challenge  During Challenge - Post-challenge  Pre-challenge - Post-challenge | .47  .93  .46 | .10  .10  .10 | < .001  < .001  < .001 |

**p* value adjustment: Tukey method for comparing a family of 3 estimates

**Table H SM.** Multilevel model predicting weekly class attendance based on baseline class attendance, instrumental beliefs, and their interactions with each challenge period. **Pre-challenge class attendance is the reference group for challenge period.**

| *Predictors* | *Estimates* [95% CI] | SE | *p* | *semipartial R^2^* [95% CI] |
| --- | --- | --- | --- | --- |
| (Intercept) | 2.73 [2.58, 2.88] | .08 | **<.001** | - |
| During-period | .86 [-.72, 1.00] | .07 | **<.001** | .06 [.04, .08] |
| Post-period | -.19 [-.33, -.05] | .07 | **.01** | .003 [.00, .01] |
| Baseline attendance | .98 [.88, 1.08] | .05 | **<.001** | .24 [.21, .27] |
| Instrumental beliefs | -.01[-.11, .09] | .05 | .87 | .00 [.00, .003] |
| During-period*Baseline | -.28 [-.38, -.18] | .05 | **<.001** | .01 [.01, .03] |
| Post-period*Baseline | -.19 [-.29, -.09] | .05 | **<.001** | .01 [.001, .02] |
| During-period*Instrumental | -.08 [-.18, .02] | .05 | .10 | .001 [.00, .01] |
| Post-period*Instrumental | -.05 [-.14, .05] | .05 | .33 | .00 [.00, .004] |
| *Random Effects* |  |  |  |  |
| σ^2^ | 1.70 |  |  |  |
| τ_00_ _PID_ | .25 |  |  |  |
| ICC | .13 |  |  |  |
| N _PID_ | 86 |  |  |  |
| Observations | 2011 |  |  |  |
| Marginal R^2^ / Conditional R^2^ | .45 / 0.52 |  |  |  |

**Table I SM.** Simple effects with estimated marginal means for each challenge period (i.e., before, during, and after) based on enjoyment motives.

| Estimated Marginal Means | Estimated marginal mean | Std  Error | 95% CI |
| --- | --- | --- | --- |
| *Low enjoyment (-1 SD)*  Pre-challenge classes  Challenge classes  Post-challenge classes | 2.67  3.67  2.41 | .11  .11  .11 | [2.46, 2.88]  [3.46, 3.88]  [2.20, 2.62] |
| *High enjoyment (+1 SD)*  Pre-challenge classes  Challenge classes  Post-challenge classes | 2.69  3.54  2.61 | .11  .10  .10 | [2.49, 2.90]  [3.34, 3.74]  [2.41, 2.82] |
| Contrasts Based on Challenge Period | Estimate | Std  Error | *p** |
| *Low enjoyment (-1 SD)*  During Challenge - Pre-challenge  During Challenge - Post-challenge  Pre-challenge - Post-challenge | .99  1.26  .26 | .10  .10  .10 | < .001  < .001  .03 |
| *High enjoyment (+1 SD)*  During Challenge - Pre-challenge  During Challenge - Post-challenge  Pre-challenge - Post-challenge | .85  .93  .08 | .10  .10  .10 | < .001  < .001  < .70 |

**p* value adjustment: Tukey method for comparing a family of 3 estimates

**Table J SM.** Multilevel model predicting weekly class attendance based on baseline class attendance, enjoyment motives, and their interactions with each challenge period. **Pre-challenge class attendance is the reference group for challenge period.**

| *Predictors* | *Estimates* [95% CI] | SE | *p* | *semipartial R^2^* [95% CI] |
| --- | --- | --- | --- | --- |
| (Intercept) | 2.73 [2.58, 2.87] | .07 | **<.001** | - |
| During-period | .91 [.77, 1.05] | .07 | **<.001** | .07 [.05, .09] |
| Post-period | -.18 [-.04, -.32] | .07 | **.01** | .003 [.00, .01] |
| Baseline attendance | .98 [.87, 1.09] | .05 | **<.001** | .23 [.20, .26] |
| Enjoyment | .01 [-.13, .15] | .07 | .89 | .00 [.00, .003] |
| During-period*Baseline | -.27 [-.37, -.17] | .05 | **<.001** | .01 [.004, .02] |
| Post-period*Baseline | -.21 [-.32, -.11] | .05 | **<.001** | .01 [.002, .02] |
| During-period*Enjoyment | -.07 [-.20, .06] | .07 | .30 | .00 [.00, .004] |
| Post-period*Enjoyment | .09 [-.05, .22] | .07 | **.21** | .001 [.00, .01] |
| *Random Effects*  σ^2^ | 1.63 |  |  |  |
| τ_00_ _PID_ | .25 |  |  |  |
| ICC | .13 |  |  |  |
| N _PID_ | 86 |  |  |  |
| Observations | 2011 |  |  |  |
| Marginal R^2^ / Conditional R^2^ | .45 /.52 |  |  |  |

**Table K SM.** Multilevel model predicting weekly class attendance based on pre-challenge weekly average class attendance, integrated regulation, and their interactions with each challenge period. **Pre-challenge class attendance is the reference group for challenge period.**

| *Predictors* | *Estimates* [95% CI] | SE | *p* | *semipartial R^2^* [95% CI] |
| --- | --- | --- | --- | --- |
| (Intercept) | 2.73 [2.58, 2.87] | .07 | **<.001** | - |
| During-period | .87 [.73, 1.01] | .07 | **<.001** | .06 [.04, .08] |
| Post-period | -.19 [-.33, -.05] | .07 | **.01** | .003 [.00, .01] |
| Baseline attendance | .98 [.87, 1.09] | .06 | **<.001** | .22 [.19, .25] |
| Integrated regulation | .02 [-.12, .17] | .07 | .75 | .00 [.00, .003] |
| During-period*Baseline | -.30 [-.41, -.19] | .05 | **<.001** | .01 [.01, .03] |
| Post-period*Baseline | -.23 [-.33, -.12] | .05 | **<.001** | .01 [.002, .02] |
| During-period*Integrated | .01 [-.12, .15] | .07 | .85 | .00 [.00, .003] |
| Post-period*Integrated | .13 [-.01, .27] | .07 | .06 | .002 [.00, .01] |
| *Random Effects* |  |  |  |  |
| σ^2^ | 1.70 |  |  |  |
| τ_00_ _PID_ | .25 |  |  |  |
| ICC | .13 |  |  |  |
| N _PID_ | 87 |  |  |  |
| Observations | 2035 |  |  |  |
| Marginal R^2^ / Conditional R^2^ | .43 / .51 |  |  |  |
